# Spontaneous Spatial Mapping of Learned Sequence in Chimpanzees: Evidence for a SNARC-Like Effect

**DOI:** 10.1371/journal.pone.0090373

**Published:** 2014-03-18

**Authors:** Ikuma Adachi

**Affiliations:** Primate Research Institute, Kyoto University, Center for International Collaboration and Advanced Studies in Primatology, Kyoto University, Inuyama, Aichi, Japan; Institut Pluridisciplinaire Hubert Curien, France

## Abstract

In the last couple of decades, there has been a growing number of reports on space-based representation of numbers and serial order in humans. In the present study, to explore evolutionary origins of such representations, we examined whether our closest evolutionary relatives, chimpanzees, map an acquired sequence onto space in a similar way to humans. The subjects had been trained to perform a number sequence task in which they touched a sequence of “small” to “large” Arabic numerals presented in random locations on the monitor. This task was presented in sessions that also included test trials consisting of only two numerals (1 and 9) horizontally arranged. On half of the trials 1 was located to the left of 9, whereas on the other half 1 was to the right to 9. The Chimpanzees' performance was systematically influenced by the spatial arrangement of the stimuli; specifically, they responded quicker when 1 was on the left and 9 on the right compared to the other way around. This result suggests that chimpanzees, like humans, spontaneously map a learned sequence onto space.

## Introduction

A growing number of studies have demonstrated that the processing of numbers and space are tightly linked. An influential paper for this line of studies was published by Dehaene and Changeux in 1993, showing that responses to relatively larger numbers are faster for the right hand, and those to smaller numbers for the left hand, even when number magnitude is irrelevant, as in a parity judgment task [1]. This effect, indicating a Spatial Numerical Association of Response Codes (SNARC effect) has since been replicated numerous times using different stimulus and task configurations (for reviews, [2]). The SNARC effect largely derives from association of numbers and extracorporeal space where smaller numbers are associated to the left in space, independent of which hand operates which button [3], [4]. Although these associations are to some extent dependent on cultural factors such as orientation of writing [5], the relationship between number and space seems to be deeply rooted in the brain's organization for these capacities; more deeply than mere cultural constructions, and they influence behavior at several levels [6].

Recent studies have tackled the underlying mechanism for this effect and so far at least three possible explanations have emerged (for reviews, [7]): 1) the functional overlap between numbers and space derives from experienced correlations between numbers and space [8], [9], 2) language has linked these two domains because of the shared vocabulary for describing spatial and numerical entities [10], 3) the link originates in general-purpose mechanisms for more recent cognitive skills such as number processing [11]. Overall, the origin of the relationship between numbers and space appears multifaceted and unlikely to be reduced to one single mechanism.

Several recent papers have provided a new perspective, hypothesizing that serial working memory position and spatial attention interact with each other to bind these two domains [7], [12], [13]. This account accords with findings that the SNARC effect is not limited to the number domain but also observed with non-numerical ordinal sequences [14]. The reported cultural difference in directionality of the SNARC effect could be explained by this perspective, because spatial attention is at least partly culturally tuned [15].

To better understand the evolutionary origin of space-based representation of numbers and serial orders in humans, the present study examined if our closest evolutionary relatives, chimpanzees show similar space-based representation to that described in humans. More specifically I examined whether chimpanzees, lacking language and cultural influences on spatial attention, process an acquired serial order based on the spatial domain, and if so, the direction of the effect.

The participating chimpanzees had learned a number sequence task in which they were exposed to multiple, randomly located Arabic numerals on a monitor and required to touch them in order from small to large [16], [17], [18]. Importantly, with the exception of one individual, named Ai [18], [19], [20], the subjects had no knowledge the meaning of these numerals; thus, the subjects only know the serial order of the numerals and no quantitative magnitude was attached to these stimuli. By focusing on the difference between Ai and the other chimpanzees, another aim was to assess any impact of magnitude representation on the effect. I compared response latencies in two test conditions in which two numerals, 1 and 9, were arranged horizontally either from left to right or right to left. The hypothesis was that if chimpanzees spontaneously associate the learned sequence with space, their performance should be systematically influenced by the stimulus arrangement. If representation of magnitude plays a significant role in shaping space-based representation, then the effect should be observed only in chimpanzee Ai.

## Methods

### Subjects and apparatus

Five chimpanzees (*Pan troglodytes*; 2 juvenile females [Pal and Cleo:11 years] and 3 adult females [Pan:27 y/o, Chloe:30y/o and Ai 34 y/o]) participated and none of them were sacrificed. The chimpanzees lived in a group of 14 individuals with access to environmentally enriched outdoor (770m2) and indoor compounds. The outdoor compound was equipped with climbing frames, small streams, and various species of trees [16], [21]. Daily meals included a wide variety of fresh fruits and vegetables fed throughout the day supplemented with nutritionally balanced biscuits (fed twice daily) and water available ad libitum. They had previously participated in a variety of computer-controlled tasks (for reviews: [20], [22]), including the number sequence task consisting of touching Arabic numerals from small to large [16], [17], [22], [23]. During the task and all training procedures the locations of the numbers were always randomized on the screen and thus subjects were trained to ignore spatial information and focus only on the sequential relationship among the numerals. The stimuli were presented on a 17-inch LCD touch panel display (1280×1024 pixels) controlled by custom-written software using Visual Basic 2010 (Microsoft Corporation, Redmond, Washington, USA). Below the display a food tray was installed in which small food rewards were delivered by a custom-designed feeder after every correct trial. Tested individually, the chimpanzees sat in an experimental booth (2.5 m wide, 2.5 m deep, 2.1 m high), separated from the experimenter by transparent acrylic panels. All procedures complied with The Guide for the Care and Use of Laboratory Primates (2002) of the Primate Research Institute, Kyoto University. The experimental protocol was approved by the Animal Welfare and Care Committee of the same institute. (protocol# 2012-090).

### Procedure

**Figure 1:**
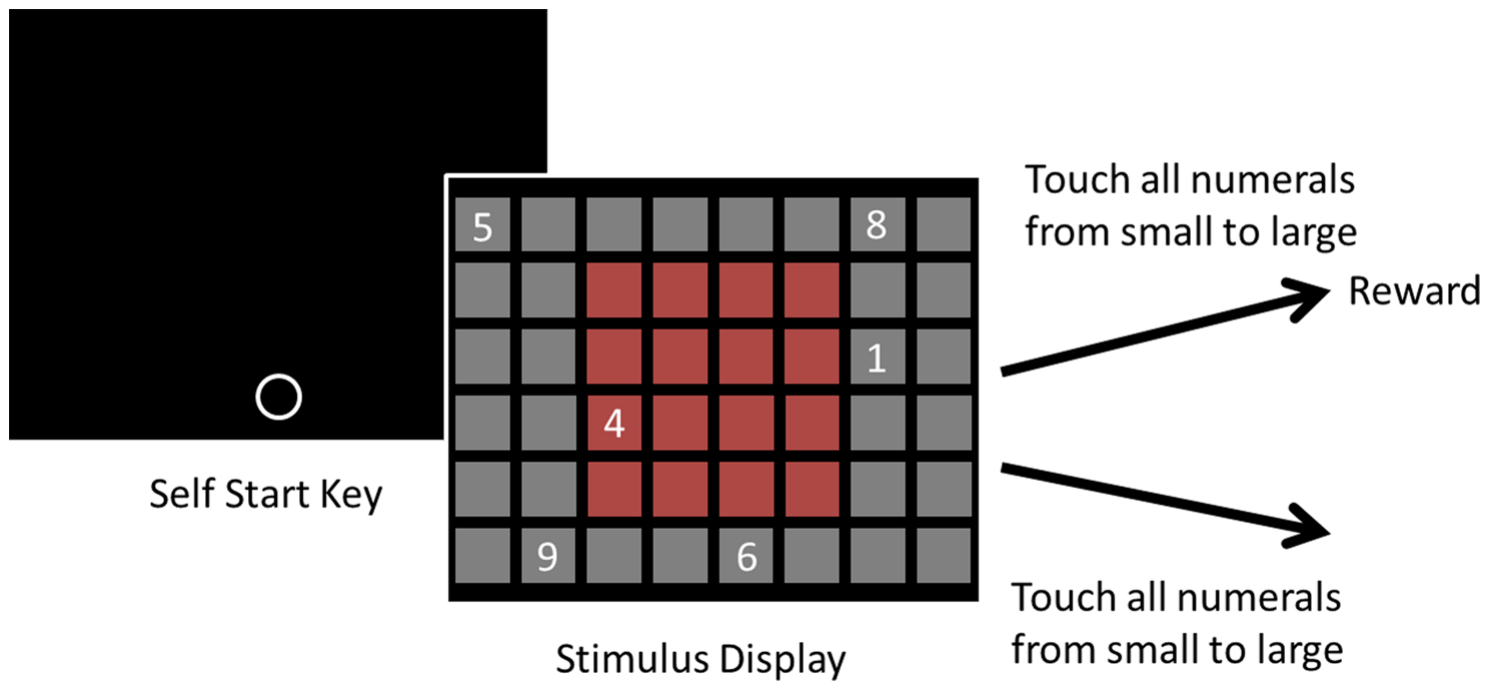
Schematic diagrams of the number sequence task. The subject's touch on the white open circle initiated a trial and random number (from 4 to 9) of Arabic numerals (baseline trials) or two numerals, 1 and 9 (test trials) were presented on the monitor. In test trials, 1 and 9 were located on the same row (either 1 on the left or 9 on the left); in baseline trials, stimuli were presented at random locations. The grid arrangement for stimulus presentations were depicted in Stimulus Display. Each grid was 130×130 pixels with a 20-pixel margin to the next grid. 4×4 grids in red were used for stimulus presentation in test trials.

After a 2-sec inter-trial interval, a white open circle was presented low down in the middle of the monitor as a self-start key (Figure 1). This was done to keep the subject's hand at the same starting location on all trials. Once the subject touched the self-start key, white Arabic numerals were presented on the stimulus field on the monitor. The stimulus field was divided into 8×6 grids (each 130×130 pixels in size and separated by 20 pixel margins, Figure 1, Stimulus Display). The numerals disappeared when the subject touched at the correct point in the sequence. When the subject touched all numerals in order from small to large, a small piece of fruit (apple or raisin) was delivered as a reward. When the subject made an error the trial was terminated and a 2-sec timeout ensued. Each session consisted of 48 trials of baseline trials and an average of 7 test trials, interspersed among the baseline trials. In the baseline trials a random sequence of between four and nine Arabic numerals (from 1 to 9) were presented in random locations on the stimulus field on the monitor. Sequences contained both adjacent and non-adjacent numerals. The chimpanzees had been trained to touch the numerals in the correct order, from small to large [16], [17]. In the test trials only two numerals (1 and 9) were presented. The locations of the two numerals were arranged horizontally. Half of the trials had 1 on the left and 9 on the right (Left-Right condition), and the other half had them the other way around (Right-Left condition) within the central 4×4 grid (grid depicted in red in Figure 1). All 48 possible horizontal arrangements within the 4×4 grids were presented and thus two numerals were presented at various possible distances. In total, 4 blocks of 48 trials (192 trials) were presented to each subject.

## Results

**Figure 2:**
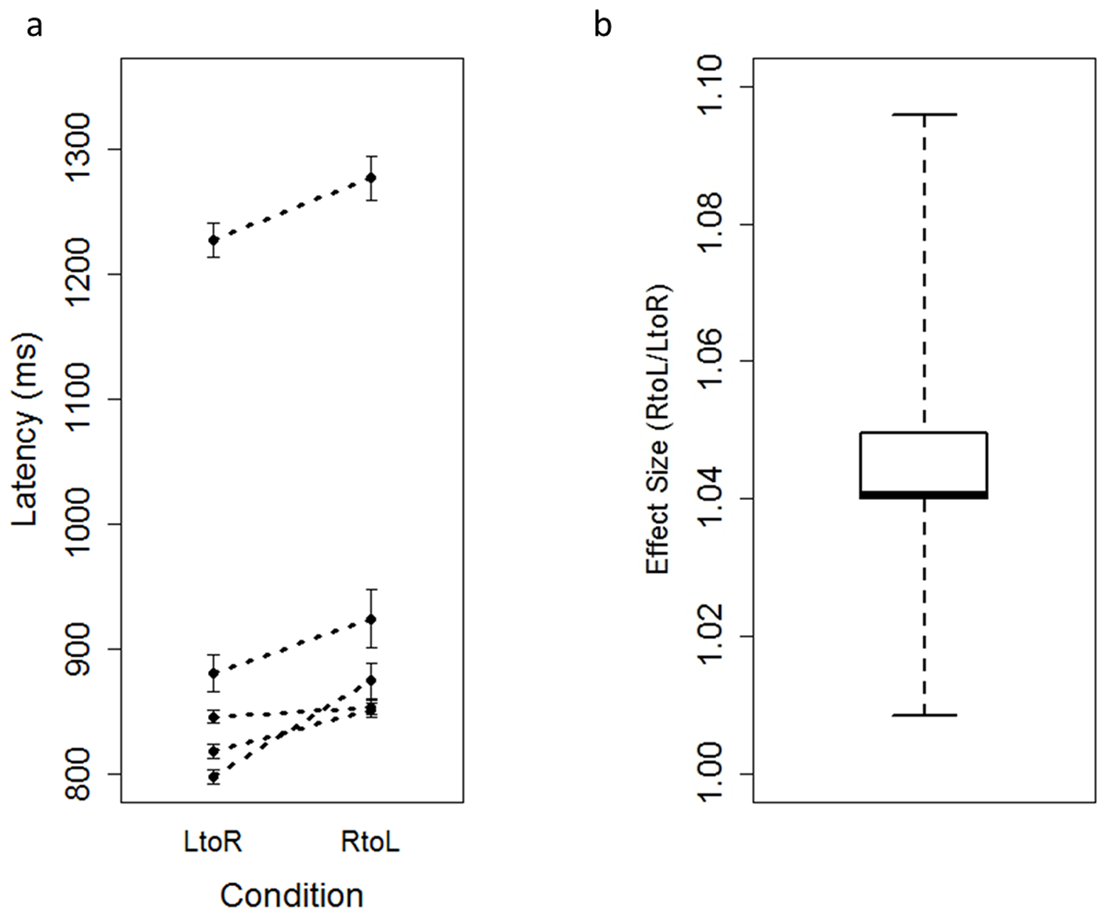
Each subject's performance. (a) Each dot represents mean latency in each condition for each subject. The dots connected by broken lines represent data from the same individuals. Error bars indicate standard error of the mean. (b) The effect size was calculated and plotted for each subject by dividing the subject's scores in Right-Left condition by that in the Left-Right condition. Any value over 1 suggests faster responding in the Left-Right condition than the Right-Left condition.

Any test trials in which the subjects made errors were excluded from the analyses (Ai: 1 trial, Chloe: 2 trials, Cleo: 5 trials, Pan: 1 trial Pal: 1 trial). The dependent variable was latency to complete the trial. First, outliers were identified for each subject with Smirnov-Grubbs outlier test and were excluded from the analyses. Given that the number of the subjects was small, paired Wilcoxon signed rank test (two-tailed) was used to compare subjects' performance on Left-Right condition and Right-Left conditions to test for any systematic bias due to the arrangement of the stimuli. The chimpanzees responded significantly faster in the Left-Right condition than the Right-Left condition (Z = −2.022, *p*<0.05, Figure 2a). I also calculated the effect size by dividing their score in the Right-Left condition by that in the Left-Right condition (Figure 2b). Their effect size were significantly bigger than 1 (Z = −2.022, *p*<0.05, a paired Wilcoxson signed rank test (two tailed)). In this Index, the chimpanzee, Ai, showed the second lowest effect and thus within the range of the other subjects.

## Discussion

The present study is the first to show that our closest evolutionary relatives, chimpanzees, share space-based representation of serial order with humans. Language is therefore not the key factor underlying such space-based representation. More likely, the space-based representation of serial order evolved for another reason. One possibility is that it might be advantageous for animals, including humans, to use analogical concrete spatial relations to process abstract concepts that cannot be directly perceived through sensory organs.

One may argue that handedness might somehow lead to an asymmetrical performance of the type observed here. The task used here required the chimpanzees to touch two items either from left to right or right to left, which might be affected by the relative ease of the kinematic hand motion. However, two chimpanzees (Pal and Chloe) used their left hand whereas the other three (Ai, Pan, Cleo) used their right hand to perform the task. Therefore, handedness can be excluded as a determining factor.

Interestingly chimpanzee Ai, who had been trained on the association between the numerals and quantity showed the second lowest effect size. Stimulus magnitude itself, therefore, seems unnecessary for formation of the observed space-based representation. There is, though, a study demonstrating that this chimpanzee, Ai, showed limited transfer between ordinal and cardinal meanings of the numerals [24]. Therefore, a remaining possibility is that using numerals in a serial ordering task may not have activated any quantity/magnitude representation very much in Ai. Further study is needed to examine this possibility. In the current study, only 1 and 9 were used in the test trials to design the simplest experiment to assess the main effect of spontaneous spatial mapping of the numerals. In the future study, however, it would be also interesting to examine an interaction between this spatial mapping and symbolic distance by using all possible combinations of these numerals. This would give us more implications on how they represent space, order, and stimulus magnitude in their brain.

It is important to recall that the numerals had been always presented at random locations on the screen during years of previous training. In other words, the chimpanzees were in fact trained to ignore spatial information while learning the sequential order of the numerals. Nevertheless, they spontaneously mapped the numerals onto the spatial domain. It thus appears that serial order processing inevitably activates spatial representation. These findings are consistent with the hypothesis that the interaction between spatial attention and serial working memory could be the factor linking space, numbers and serial orders. Moreover, it is interesting that the directionality of the spatial mapping of the serial order was left-right in chimpanzees. This is not inconsistent with most of the previous studies that showed we humans associate small numbers to the left in space (see review [2]). This may imply that left-right spatial mapping of serial orders is biologically determined and is more likely than the converse. To better understand what the biological basis of this directionality is, further studies are required to explore underlying mechanisms.

Together with the previous literature, the present study has the following implications for the space-based representation. First, language is not the fundamental factor for space-based representation of numbers and serial order. Probably, such representation has evolved to enhance understanding of abstract concepts, such as time and order, that cannot be directly perceived through our sensory organs. This is achieved by encoding these abstract concepts within a more concrete domain such as space. Second, the default direction of this space-based representation is from left to right, although culture and language might modify this to some extent. Third, the space-based representation of numbers stems from the space-based order of serial order. Conceivably, interaction between working memory for these orders and spatial attention provides a foundation for space-based representation. Ordinal information is embedded in numbers, and so space-based representation can be easily applied for numbers as well.

## References

[1] Dehaene S, Changeux JP (1993) Development of Elementary Numerical Abilities - a Neuronal Model. Journal of Cognitive Neuroscience 5: 390–407. 23964915 10.1162/jocn.1993.5.4.390

[2] Fias W, Fischer MH (2005) Spatial representation of number. In: Campbell JID, editor. Handbook of Mathematical Cognition. East Sussex: Psychology Press. pp. 43–54.

[3] Müller D, Schwarz W (2007) Is there an internal association of numbers to hands? The task set influences the nature of the SNARC effect. Memory & Cognition 35: 1151–1161. 17910196 10.3758/bf03193485

[4] Fischer M (2003) Spatial representations in number processing–evidence from a pointing task. Visual Cognition 10: 493–508.

[5] Shaki S, Fischer MH (2008) Reading space into numbers: a cross-linguistic comparison of the SNARC effect. Cognition 108: 590–599. 18514179 10.1016/j.cognition.2008.04.001

[6] Hubbard EM, Piazza M, Pinel P, Dehaene S (2005) Interactions between number and space in parietal cortex. Nature Reviews Neuroscience 6: 435–448. 15928716 10.1038/nrn1684

[7] Fias W, van Dijck J-P, Gevers W (2011) How is Number Associated with Space? The Role of Working Memory. In: Brannon SDaE, editor. Space, Time and Number in the brain: Elsevier. pp. 133–148.

[8] Dehaene S, Bossini S, Giraux P (1993) The mental representation of parity and number magnitude. Journal of Experimental Psychology: General 122: 371–396.

[9] Chen Q, Verguts T (2010) Beyond the mental number line: A neural network model of number–space interactions. Cognitive Psychology 60: 218–240. 20138261 10.1016/j.cogpsych.2010.01.001

[10] Casasanto D, Boroditsky L (2008) Time in the mind: Using space to think about time. Cognition 106: 579–593. 17509553 10.1016/j.cognition.2007.03.004

[11] Dehaene S, Cohen L (2007) Cultural Recycling of Cortical Maps. Neuron 56: 384–398. 17964253 10.1016/j.neuron.2007.10.004

[12] van Dijck JP, Abrahamse EL, Majerus S, Fias W (2013) Spatial attention interacts with serial-order retrieval from verbal working memory. Psychol Sci 24: 1854–1859. 23863755 10.1177/0956797613479610

[13] van Dijck J-P, Fias W (2011) A working memory account for spatial–numerical associations. Cognition 119: 114–119. 21262509 10.1016/j.cognition.2010.12.013

[14] Gevers W, Reynvoet B, Fias W (2003) The mental representation of ordinal sequences is spatially organized. Cognition 87: B87–B95. 12684205 10.1016/s0010-0277(02)00234-2

[15] Maass A, Russo A (2003) Directional Bias in the Mental Representation of Spatial Events: Nature or Culture? Psychological Science 14: 296–301. 12807400 10.1111/1467-9280.14421

[16] Tomonaga MMT, Itakura S (1993) Teaching ordinals to a cardinal trained chimpanzee. Primate Research 9: 67–77.

[17] Inoue S, Matsuzawa T (2007) Working memory of numerals in chimpanzees. Curr Biol 17: R1004–1005. 18054758 10.1016/j.cub.2007.10.027

[18] Matsuzawa T (2009) Symbolic representation of number in chimpanzees. Current Opinion in Neurobiology 19: 92–98. 19447029 10.1016/j.conb.2009.04.007

[19] Matsuzawa T (1985) Use of Numbers by a Chimpanzee. Nature 315: 57–59. 3990808 10.1038/315057a0

[20] Matsuzawa T (2003) The Ai project: historical and ecological contexts. Animal Cognition 6: 199–211. 14566577 10.1007/s10071-003-0199-2

[21] Matsuzawa T (2006) Sociocognitive Development in Chimpanzees: A synthesis of Laboratory Work and Fieldwork. In: Matsuzawa T, Tomonaga, M, Tanaka, M., editor. Cognitive Development in Chimpanzees. Tokyo: Springer. pp. 3–33.

[22] Matsuzawa T, Tomonaga M, Tanaka M (2006) Cognitive Development in Chimpanzees: Springer.

[23] Kawai N, Matsuzawa T (2000) Numerical memory span in a chimpanzee. Nature 403: 39–40. 10638743 10.1038/47405

[24] Biro D, Matsuzawa T (2001) Use of numerical symbols by the chimpanzee (Pan troglodytes): Cardinals, ordinals, and the introduction of zero. Animal Cognition 4: 193–199. 24777509 10.1007/s100710100086

